# Accelerated Method for Determining the Fatigue Limit of Trabecular Bone

**DOI:** 10.3390/ma18020232

**Published:** 2025-01-08

**Authors:** Artur Cichański, Tomasz Topoliński, Krzysztof Nowicki

**Affiliations:** Faculty of Mechanical Engineering, Bydgoszcz University of Science and Technology, Kaliskiego 7, 85-796 Bydgoszcz, Poland

**Keywords:** bone fatigue, accelerated test, trabecular bone, mechanical properties, bone structure indices

## Abstract

This paper presents an experimental method for estimating the fatigue limit of trabecular bone using a single trabecular bone sample, the microstructural parameters of which were determined by microCT. Fatigue tests were carried out using the Locati method, with stepwise increasing load amplitude. The fatigue limits of the trabecular structures were determined experimentally in accordance with Miner’s law of fatigue damage accumulation, based on the parameters of the reference S-N curve taken from the literature. On the basis of the fatigue limits, the S-N curves were determined for the tested samples, and from them the compressive strength *US_S-N_* corresponding to the fatigue limit for the *N* = 1 cycle. Ultimate compressive strength *US* was determined as a result of compression to failure tests. Computational dependencies combining the *BV/TV* index with *US* and the *BMD* index with *US* were formulated. To verify the proposed method, two groups of human trabecular bone samples were analysed: *n* = 42 were tested under monotonic loading, and *n* = 61 were tested under cyclic loading with stepwise increasing amplitude. The statistical test of the distribution conformity of the calculated *US_S-N_* compressive strength to the experimental *US* ultimate strength was performed. The results of the Kolmogorov–Smirnov statistical test were *D* = 0.19 (*p* = 0.314). The agreement of the distributions of *BV/TV*, as determined experimentally and calculated from the computational dependencies, was also tested statistically, with the result of the Kolmogorov–Smirnov test being *D* = 0.286 (*p* = 0.065). A similar analysis performed for *BMD* yielded *D* = 0.238 (*p* = 0.185).

## 1. Introduction

The majority of human fractures are caused by trauma. Additionally, bone fractures may result from fatigue processes within bone tissue that occur under cyclic loading conditions due to prolonged repetition of specific activities. In order to test fatigue under laboratory conditions, material specimens are subjected to cyclic loading, which is usually of a sinusoidal nature. The application of such loads results in the generation of cyclic stresses within the tested material, with an amplitude (S) that corresponds to a specific number of cycles leading to damage (N). As the load amplitude increases, the number of cycles to failure decreases. This regularity is illustrated in graphical form by the S-N curve [1]. Fatigue life prediction for construction materials is based on the availability of the S-N curve. There is a characteristic point on the S-N curve, which is the fatigue limit *σ_f_* corresponding to the assumed fatigue life *N_f_*.

Bone tissue is spatially distributed within the volume of the cancellous bone to form trabecular structures. Bone strength is influenced by the number and thickness of the trabeculae and their spatial orientation in relation to the external load. During fatigue testing of bone samples, the mechanical properties of the trabecula do not change, but its damage weakens the entire tissue structure. Mechanical testing of individual trabecula is performed at the micro scale. A two-point bending test [2] of a single trabecula observed the first quasi-static crack at 1N force. During the tension-compression test of a single trabecular [3], the displacement amplitude was 50 µm and the first microcracks were observed at 1500 cycles. Tests of the fatigue strength of the bone structure are conducted on a macro scale [4].

S-N curves are determined in the whole bone test [5], but mainly on cortical bone [4,6,7] and trabecular bone [8,9,10] specimens. Bone fatigue tests are usually performed with periodically varying stresses with a sine waveform [3,10,11,12], a half-sine waveform [13] or a haversine waveform [11]. Tests are usually performed at a frequency of 2 Hz [4,5,11,14], and less frequently at 0.5 Hz [4], 1 Hz [3], 3 Hz [15], 4 Hz [10] and 5 Hz [12]. Sine waveform tests are most often performed with the upper fatigue limit of 10^6^ cycles [6,16], but there are studies where tests were completed with significantly fewer cycles [17]. In all cases, the upper limits of fatigue life and the low frequency resulted in increased test time and cost.

The S-N curves obtained from the tests are presented in a semi-logarithmic coordinate system Δ*σ*-log*N_f_* [18], *σ*-log*N_f_* [6] or *S*-log*N_f_* [19]. Sometimes the results are presented in a bi-logarithmic coordinate system—logΔ*σ*/E_0_-log*N_f_* [16], log*σ*/*E*_0_-log*N_f_* [7,8,9] or log*S*-log*N_f_* [19,20]—where Δ*σ* represents the range of nominal stress changes, *σ* is the stress amplitude or maximum nominal stress (minimum for compression), *E*_0_ represents the initial secant module, *S* is the force load and *N_f_* is the fatigue life. The stress coordinate of the S-N plot determined using the modulus *E*_0_ is defined as the normalised change in the stress range (respectively Δ*σ*) or normalised stress (respectively *σ*). For stress-controlled tests, the ratio Δ*σ*/*E*_0_ or *σ*/*E*_0_ is the unspecified value of the change in the range of the initial strain or initial strain that remains unchanged during the test. In [21] the ratio *σ*/*E*_0_ is defined as the initial global strain. Introducing the modulus *E*_0_ into the stress coordinate in the S-N curve is a way of obtaining consistent results based on the assumption that bone architecture mainly affects the initial secant modulus *E*_0_ [22,23,24,25]. In this situation, *E*_0_ must be determined for each specimen prior to the fatigue test.

Ref. [26] presents the method for determining *E*_0_, which consists in cyclically deforming the specimen in the range from 0% to 0.2% under a load of 10 N at a frequency of 2 Hz for 10 cycles to determine the modulus value in the last cycle. The initial modulus *E*_0_ as the secant modulus was determined using the similar method w [8,27]. The determination of the initial modulus *E*_0_ as the tangent modulus in progressive loading fatigue tests on trabecular bone specimens has been described and verified by the authors in [28]. In the first stage of the calculations, the best fit line was determined for the upper branch of the *σ*-*ε* hysteresis loop. The fit was performed in the range of 85% to 100% of the maximum stress of the loop. The tangent of the slope of the best fit line therefore determined the initial modulus *E*_0_. Due to the need for experimental determination of *E*_0_ in the fatigue test and the wide range of variability in the factors influencing the determination of S-N curves defined using *S*/*E*_0_, the effectiveness of the curve modified in this way is limited.

The coefficient of determination R^2^ obtained in the analysis of the results of fatigue tests with a normalised *E*_0_ range of stresses or strains ranges was 0.54 [27]–0.946 [17] for human trabecular bone, 0.52 [9]–0.86 [8] for bovine trabecular bone and 0.82 [7] for human cortical bone. Maximum R^2^ values have been reported for samples from nine donors [8]. Most studies analysed samples from 1 to 19 donors [29]. The number of human bone samples used to determine the S-N curves was a maximum of 52 (11 donors) [27] and a minimum of 8 from individual donors [30].

The aim of the study was to evaluate the possibility of accelerating the determination of the fatigue limit of the trabecular bone during the fatigue test performed using the stepwise increasing load amplitude and parameters of the basic S-N curve for the bone material adopted from the literature. The advantage of this method is not only the reduction of research time, but also the fact that the experiment can be carried out with only one sample. The statistical verification of the presented method was performed using computational relationships connecting selected indices of trabecular bone structure to ultimate strength. The regression models obtained in this work can be used in practice to estimate the fatigue limit of the bone structure whose indices have been determined in vivo.

## 2. Materials and Methods

### 2.1. Trabecular Bone Samples

The samples were obtained by cutting them from the heads of human femoral bones obtained after femoral joint prosthesis implantation. The protocol was approved by the Ethics Committee of Nicolaus Copernicus University CM, approval no. KBp 7/2024. The inclusion criterion for the study was a minimum age of 45 years. The eligibility criterion for hip arthroplasty was hip joint dysfunction. Due to the invasive nature of the procedure, general health was indirectly taken into account. Only people whose general condition was suitable for the procedure were eligible, e.g., they had good blood clotting and were not significantly overweight, which would make general anaesthesia difficult. The study used two sample groups. The two groups did not differ in terms of gender or age. There was an equal representation of women and men, which allowed us to exclude the influence of this factor on the results.

**Figure 1:**
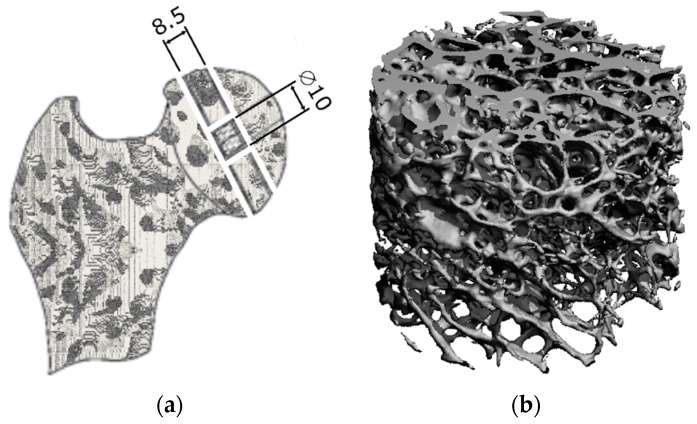
Method of obtaining samples: (**a**) sample cutting scheme; (**b**) visualisation of trabecular bone structure BV/TV = 0.133, BMD = 0.206.

From the thick slices cut from the femoral heads perpendicular to the axis of the neck of the bone, cylinders 10 mm in diameter and 8.5 mm in length were drilled using a self-designed core drill (Figure 1a). Due to the sizes of the specimens, it was only possible to prepare one for each patient.

All samples were divided into two groups, as presented in Table 1. Group I of the samples was used to verify the model, the parameters of which were determined during the calibration carried out on the samples of Group II. The statistical tests carried out showed no significant differences between the two groups of samples. Samples were stored in 10% formalin solution at room temperature. Storing the bone samples in formalin did not significantly affect the elastic properties of the trabecular bone, as was determined in [31].

### 2.2. Micro CT Technique and Densitometry

All specimens were scanned with a desktop micro-CT system using the μCT-80 system (SCANCO Medical AG, Bruettiselllen, Switzerland). The specimens were scanned with the following parameters: 70 kV, 114 μA, 500 projections/180° and 300 ms integration time at a resolution of 36 μm. The scanning resolution chosen was sufficient to correctly represent the stiffness of the trabecular structure, as verified by the authors in µFE analyses [32], In these analyses, the apparent stiffness correlates well (R^2^ = 0.888) with the mean volume of the scanned layer at a resolution of 36 μm. The increase in voxel size of the reconstructed trabecular bone structure corresponding to the decrease in scan resolution results in an underestimation of apparent stiffness [33]. The visualisation of an example trabecular structure after reconstruction from scanned images is shown in Figure 1b. After scanning, the values of 11 bone structure indices were obtained utilising microCT software (a dedicated software for the μCT-80 system) that used algorithms based on the work of [34]. For the samples tested, the following indicators were determined: volume of trabecular tissue *BV*, total volume of specimen *TV*, volume fraction *BV/TV*, area of surface of the trabecular tissue *BS*, surface-to-volume ratio of bone *BS/BV*, average trabecular thickness *Tb.Th*, average distance between trabeculae *Tb.Sp*, average number of continuous trabeculae per unit volume of specimen *Tb.N*, number of connections between individual trabeculae per unit volume of specimen *Conn.D*, index defining type of architecture of the specimen *SMI* and degree of structural anisotropy *DA*. Bone mineral density *BMD* was measured using the Expert LUNAR (Lunar Corporation, Madison, WI, USA) densitometer.

### 2.3. Mechanical Tests

**Figure 2:**
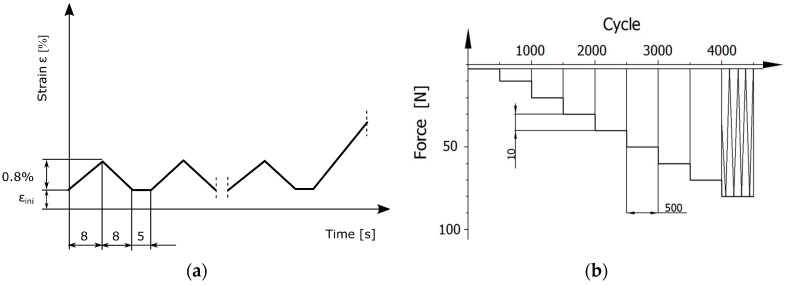
Loads scheme: (**a**) Initial compression test; (**b**) Fatigue test: minimum loading 5 N, maximum loading started from 10 N with 10 N load step, 500 cycles of each load level.

The sample of the tested bone was axially compressed between two polished plates. The standardisation of test conditions for both groups of specimens included an initial compression test with 5 cycles controlled by strain (Figure 2a). The interval between cycles was 5 s. The initial cycles were aimed at stabilising the interface between the specimen and the holder surface. The initial compression test procedure started with the determination of *ε_ini_* corresponding to a preload of 50 N. The value of *ε_ini_*, depending on the structure of the specimen, was different for each specimen. During the initial compression, the strain changes from *ε* = *ε_ini_* to *ε* = *ε_ini_* + 0.8% with a triangular waveform (Figure 2a).

Compression to failure tests were performed on Group I specimens. A maximum compressive load was used to determine the ultimate compressive strength *US* (ultimate compressive strength of trabecular tissue, [MPa]). Compressive strength was determined using the MTS (Minneapolis, MN, USA) testing machine Mini Bionix 858. Both compression tests, initial and failure tests, were performed at a strain rate of 0.001 1/s.

**Figure 3:**
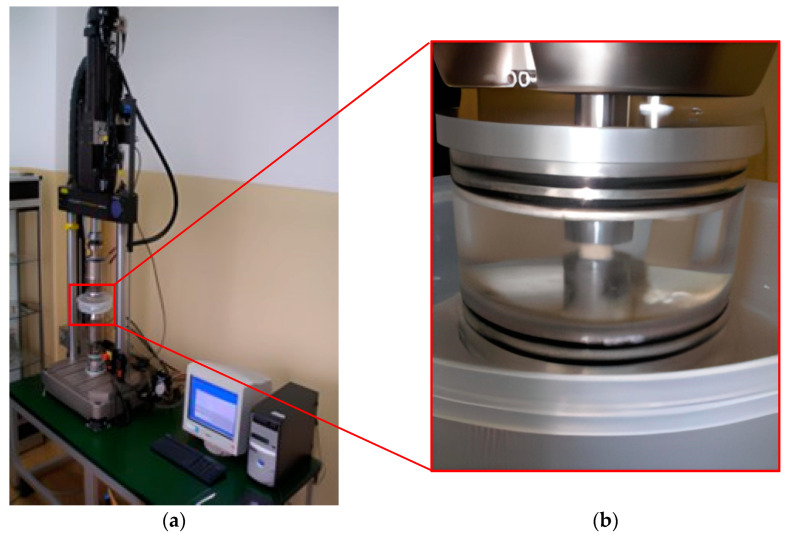
Test stand (**a**) general view; (**b**) enlarged section of test chamber with sample.

Fatigue tests were carried out on Group II specimens. Fatigue tests were carried out under cyclic compression using the INSTRON 8874 testing machine (Instron, Norwood, MA, USA). The load amplitude increase scheme for the fatigue test, defined as stepwise increasing load amplitude, is shown in Figure 2b. The minimum load for this scheme was 5 N. The maximum load started at 10 N and was increased in increments of 10 N after 500 load cycles. The frequency of the sinusoidal loading was 1 Hz. The number of cycles for each load amplitude was determined based on the results of experiments on steels and polymer composites to achieve a minimum of 6 load amplitude levels for each specimen [35]. The criterion used to determine the specimen failure was the increase in deformation of the specimen. In subsequent cycles on each load amplitude, the values of deformation increment were determined and its median was calculated. The test ended when the deformation increase in the cycle was greater than 10% of the median calculated for the load level. As a result of the fatigue tests, the maximum stress *σ_max_* was determined for the last step of the stepwise load amplitude increase. In addition, the fatigue life *N_step_*, which is the total number of cycles achieved throughout the test, was recorded. All fatigue tests were performed in an acrylic chamber containing 0.9% NaCl solution at a temperature of 37 ± 2 °C (Figure 3b).

### 2.4. Fatigue Limit Estimation

#### 2.4.1. Miner’s Law

The process of fatigue bone damage is initiated by the formation of microcracks, which subsequently progress through the crack growth phase, ultimately resulting in a fracture. At the initial stage of bone fatigue fracture development, diagnosis can be achieved through magnetic resonance imaging and computed tomography [36]. In order to describe the process of crack initiation, it is assumed that each stress cycle whose amplitude *σ_i_* is greater than the limiting value *σ_f_* contributes to the generation of material damage. The occurrence of damage *D_i_* in a material is dependent upon the number of stress cycles *σ_i_* experienced, with a crack forming when the number of cycles reaches a threshold value *N_i_* according to the following dependency: *D_i_* = *n_i_*/*N_i_*. In conditions of loads of varying amplitudes, damage is accumulated in accordance with the following relationship:

(1) $$\sum \frac{n_{i}}{N_{i}}=D,$$

where

*n_i_* – number of cycles performed with a stress amplitude *σ_I_*,

*N_i_* – fatigue life, that can be expected to lead to failure under a stress amplitude *σ_I_* and

*D* – damage function.

The linear fatigue damage accumulation according to Miner’s law [37] states that fatigue fracture occurs when the cumulative damage reaches unity (*D* = 1). According to this law, the initial state of the specimen for *n_i_* = 0 is characterised by the value of the damage function *D* = 0, and at specimen failure the value of the damage function is *D* = 1. In the cumulation operation (1), only cycles with stress amplitudes *σ_i_* > *σ_f_* are considered. The hypothesis of damage cumulation can be applied to bone material, as microscopic analysis has confirmed the occurrence of microdamage as a result of application load cycles [38]. On a macro scale, a decrease in the stiffness of the trabecular structure is observed with an increase in the number of fatigue cycles [39].

#### 2.4.2. Locati Test Method

The Locati test method was used to estimation of the fatigue limit *σ_f_* of each specimen [40]. In this method, the reduction in test time is achieved by using a stepwise increasing load amplitude (Figure 2b) rather than a constant amplitude. The fatigue limit approximation in the Locati method assumes that the slope of the reference S-N curve is known in advance. Further assumptions are that the number of cycles for each load amplitude is fixed and that the amplitude increment is also fixed. To describe the fatigue properties of trabecular bone, the form of a generalised S-N curve has been adopted:

(2) $$\frac{\sigma }{E_{0}}=aN^{b}$$

where

*a* – intercept,

*b* – slope of S-N curve,

*σ* – fatigue strength,

*N* – fatigue cycles and

*E*_0_ – initial stiffness modulus.

A position of the reference S-N curve (2) was assumed in the study based on data from the literature. The average initial stiffness *E*_0_ = 251 MPa was assumed based on the test results [17]. The slope coefficient *b* = −0.1094 was assumed based on the analysis performed by authors [41]. The analyses performed included a variant of the constant value of the *a*-intercept of the S-N curve and a variant of the constant value of the *b*-slope of the S-N curve. In both variants, the value of the damage function *D* = 1 was assumed. The fatigue life threshold of *N_f_* = 10^6^ cycles was adopted as the fatigue life used in bone fatigue tests [8,27,42].

**Figure 4:**
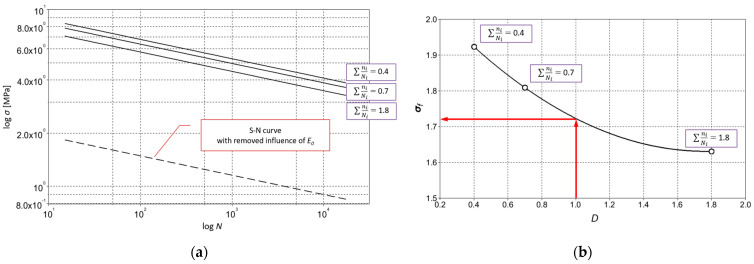
Locati method scheme: (**a**) Diagram showing the arrangement of S-N curves for the assumed values of *a_i_*; (**b**) Method of determining *σ_f_* value for *D* = 1 based on quadratic interpolation of working values of damage function *D_i_*.

Three curves are assumed, described by assumed values of *a*_1_, *a*_2_, *a*_3_ and the *b*-slope of the material S-N curve (Figure 4a). The assumed values of the intercept *a_i_* were responsible for the vertical shift of the S-N fatigue curve (Figure 4a). These curves have three points of intersection with the progression of the stepwise increase in amplitude determined in the experiment, which ended at the value of the maximum stress *σ_max_*. Each point of intersection with an assumed *a_i_* curve determined the value of *σ_i_* corresponding to the damage *D_i_* calculated by Miner’s Law. Based on the value of *D_i_* and the corresponding values of *σ_i_*, a second degree polynomial *σ_i_*-*D_i_* was constructed (Figure 4b). Under the assumptions of the linear hypothesis of fatigue damage accumulation, the *σ_f_* value for a given specimen was read from the interpolation polynomial for the value of *D* = 1 (Figure 4b).

After introducing *σ_f_* into the relationship (2) and transforming it with respect to the coefficient *a*, the following relationship is obtained:

(3) $$a=\frac{\sigma_{f}}{E_{0}N_{f}^{b}}$$

On the basis of the determined fatigue limit value *σ_f_* and the assumed values of other variables, the value of the coefficient *a* was determined from the relationship (3). The determined values of the intercept *a* and the fatigue limit *σ_f_*, together with the assumed values of *E*_0_, *b* and *N_f_,* provide a complete description of the S-N curve for a given specimen.

### 2.5. Statistics

Pearson’s correlation coefficients were used to describe the relationship between bone fatigue test results and chosen microarchitecture indicators, with an assessment of the probability of significance of this correlation. The Shapiro–Wilk method was used to test the normality of the distribution of the bone structure indices. For those bone structure indices whose distribution was incompatible with normality, the Kolmogorov–Smirnov method was used to test the goodness of fit of both distributions. The two-sample Kolmogorov–Smirnov test was chosen because it is sensitive to differences in both the location and shape of the empirical cumulative distribution functions of the two specimens. All statistical calculations were performed in R software version 3.4.3 [43].

### 2.6. Structure Indices Selected for Tests

Preliminary statistical analyses were carried out on the structural indices obtained for Group I samples [22]. The PCA tests performed and the correlation matrices obtained showed statistical significance for the BV/TV index, regardless of the type of analysis. The *BMD* index was also selected for further consideration. Bone mineral density (*BMD*) is one of the most commonly used indicators of fracture risk [44]. This indicator is measured using the Dual-Energy X-ray Absorptiometry DXA method. The wide availability of densitometry equipment makes it a commonly used indicator of bone health, although it underestimates the risk of fracture. One of the reasons for this may be the lack of reference to bone microarchitecture [45]. This defect is not shown by *BV*/*TV,* the second of the selected indices. This indicator is determined by the micro-computed tomography μCT method [46]. The BV/TV index, defined as the volume of mineralised bone fraction per unit volume of sample, describes the degree of filling of the bone volume by the mineral fraction. The *BV*/*TV* index shows a high correlation (R^2^ = 0.946) with the apparent stiffness modulus for bone [47]. This method is limited to small samples and in vitro tests. In vivo determination of the *BV*/*TV* ratio in living tissue is possible using high-resolution peripheral quantitative computed tomography (HR-pQCT). Bone volume fraction measured by HR-pQCT correlated well with μCT measures R^2^ = 0.86 [48].

## 3. Results

### 3.1. Statistical Verification of Structural Indices

As this study used two independent groups of trabecular bone specimens, it was necessary to verify that they belonged to the same population. Table 2 shows the descriptive statistics of the selected structural indices for both groups of samples.

The consistency of the *BMD* and *BV/TV* structural indicators used in the study was statistically verified. First, it was checked whether the statistical distribution of selected structural indicators calculated independently for each of the two specimen groups was consistent with the normal distribution. The Shapiro–Wilk test was used to check this conformity. The values of the critical statistic *W* and the *p*-value for selected indicators are presented in Table 3.

For both indicators, the *p*-value in Group I is lower than in Group II (Table 3). Since the *p*-value for the *BMD* indicator of Group I specimens is less than the 0.05 confidence level, the distribution of the probability density for this indicator is not compliant with the normal distribution. Since not all the structural indices in the paper conform to the normal distribution, a non-parametric Kolmogorov–Smirnov test was used to check the conformity of their distribution for both sample groups. In the first test, the values of *BV/TV_I_* and *BV/TV_II_* were compared, and in the second test, the values of *BMD_I_* and *BMD_II_* were compared. The Kolmogorov–Smirnov statistic *D* and the *p*-value are shown in Table 4.

The *D*-statistics and *p*-values allow this study to advance the hypothesis that both specimen groups are from the same population based on the *BV/TV* values (Table 4). There is no statistical basis for a similar statement regarding the *BMD* values. The authors of [49] discuss the low reliability of *BMD* in assessing bone quality. Using µFE analyses performed on samples of trabecular structure, a compressive force to induce strain *ε* = 0.8% was determined. A compressive force correlates well (R^2^ = 0.9) with the mean value of the fractal dimension of all microCT scanned sample layers. A low correlation of R^2^ = 0.5 was obtained for the relationship between compressive force and *BMD*.

### 3.2. Statistical Verification of Test Results

Ultimate compressive strength *US* was determined as a result of static compression tests on Group I specimens. Group II specimens were subjected to fatigue compression tests. As a result of these tests, the maximum stress *σ_max_* and the total number of cycles to failure *N_step_* were determined. The descriptive statistics of the mechanical test results for both groups of specimens are presented in Table 5.

Similarly to the indicators, the test results were checked to see if their statistical distribution was consistent with a normal distribution. Table 6 shows the results of the Shapiro–Wilk test for specimens from both groups.

Since the *p*-value is below the 0.05 confidence level, the hypothesis of normality of the probability distribution of the experimental results analysed cannot be accepted. The comparison of the results of the calculations will be carried out by means of the non-parametric statistical test Kolmogorov–Smirnov.

### 3.3. Assumption for the Verification of Model

Due to the inability to obtain the required number of specimens from a single donor, it was not possible to experimentally verify the S-N curve determined by the Locati method on specimens of the same bone structure. Under these conditions, it was assumed that it would be possible to use a method in which the comparison was made using the compressive strength calculated from the fatigue curve and the *US* determined in static tests on specimens from different bones.

#### 3.3.1. Assumption for Comparison of US-USS-N

It has been assumed that it is possible to indicate on the S-N curves defined for such stresses comparable to the ultimate compressive strength determined in compression-to-failure tests. The verification of such an assumption consists of checking whether the calculated values of the compressive strength *US_S-N_*, obtained from the results of the fatigue tests, and the experimental values of the ultimate compressive strength *US*, obtained from the static tests, are statistically consistent. This approach is consistent with the previous statement, based on the results of the Kolmogorov–Smirnov test, that both groups of samples were selected from the same population based on *BV/TV* index values (Table 4).

#### 3.3.2. Assumption for Comparison of BV/TV-BV/TV_cal_ and BMD-BMD_cal_

In addition to a direct comparison of the two sets of ultimate compressive strengths, it was proposed to verify whenever the *US_S-N_* compressive strength was representative of the fatigue test results. Indirect verification involved the use of computational and experimental indicators of the structure of individual specimens. This required the construction of auxiliary models combining the *BV/TV_II_* and *BMD_II_* indicators with the *US_S-N_* compressive strength calculated from the fatigue test results for Group II specimens.

### 3.4. Verification of Fatigue Limit Estimation

#### 3.4.1. Comparison of US-US_S-N_

**Figure 5:**
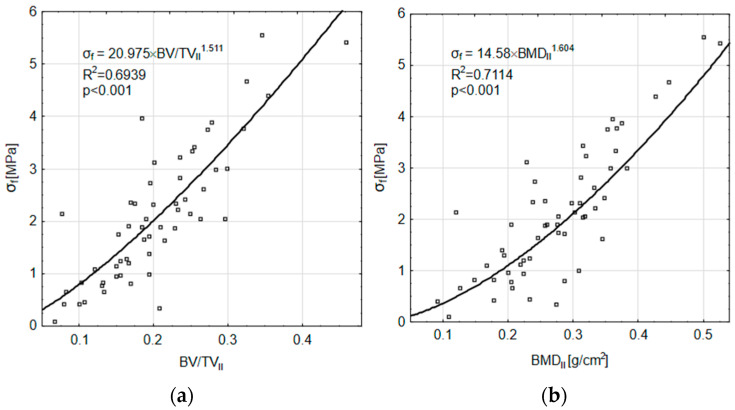
Fatigue limit *σ_f_*: (**a**) Related to *BV/TV_II_*; (**b**) Related to *BMD_II_*.

The fatigue tests determined the maximum stress *σ_max_* and the fatigue life *N_step_* allowed for the calculation of the fatigue limit *σ_f_* using the Locati method. Figure 5a shows the relationship between the fatigue limit *σ_f_* and the *BV/TV_II_* index. For this relationship the R^2^ = 0.6939 was obtained and the CI confidence interval ranged from 0.4935 to 0.7872; *p* < 0.001. Figure 5b shows the relationship between *σ_f_* and the *BMD_II_* index value. For this relationship, the R^2^ = 0.7114 was obtained and the CI confidence interval ranged from 0.4032 to 0.7349; *p* < 0.001.

The S-N curve illustrates the increase in fatigue life with the decrease in fatigue strength interpreted as the amplitude of the fully reversed fatigue cycle. In this paper, *US_S-N_* compressive strength has been calculated as the fatigue limit corresponding to *N* = 1 fatigue life from the S-N curves for a given specimen. Such determined the *US_S-N_* values refer to the maximum stress acting in ¼ of the fatigue cycle, the stress corresponding to this cycle amplitude. The descriptive statistics of the fatigue limit *σ_f_* and compressive strength *US_S-N_* calculated for Group II specimens are presented in Table 7.

**Figure 6:**
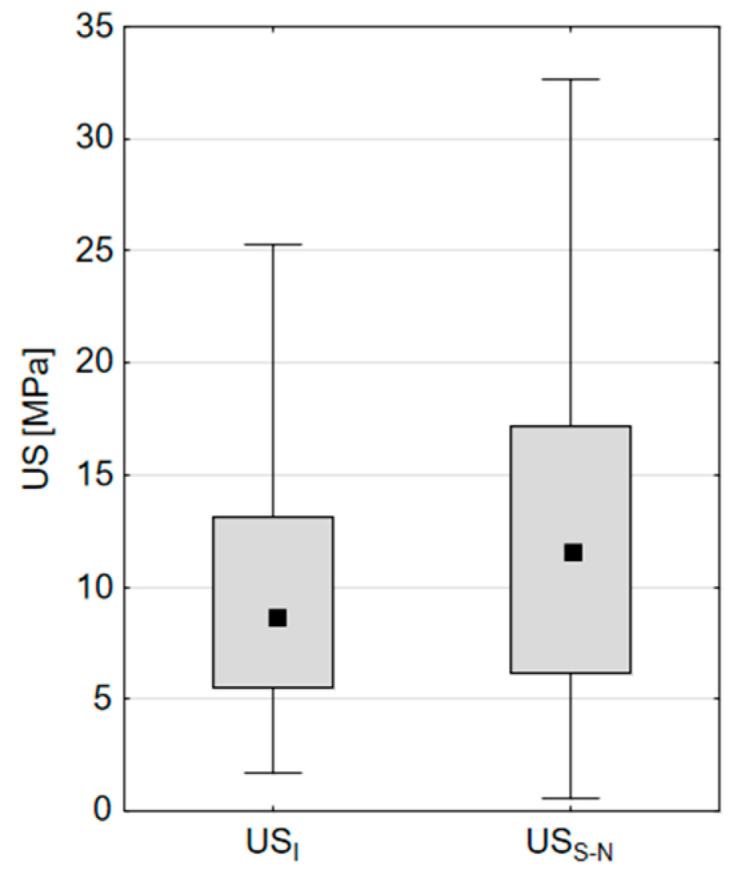
Comparison of experimental *US_I_* and calculated *US_S-N_*.

The calculated values of *US_S-N_* were compared with the experimental values of *US_I_* using a box plot, as shown in Figure 6. The Kolmogorv–Smirnov test was used to statistically verify the assumption about *US_S-N_*. This verification required the statistical test of the distribution agreement between the calculated values of the compressive strength *US_S-N_* (Table 7) obtained for the Group II specimens using S-N curves and the experimental values of the ultimate compressive strength *US_I_* obtained for the Group I specimens (Table 5). The statistical test results of *D* = 0.19 and *p*-value = 0.314 show that the hypothesis of agreement of the distribution of both values cannot be rejected.

#### 3.4.2. Comparison of BV/TV-BV/TV_cal_ and BMD-BMD_cal_

**Figure 7:**
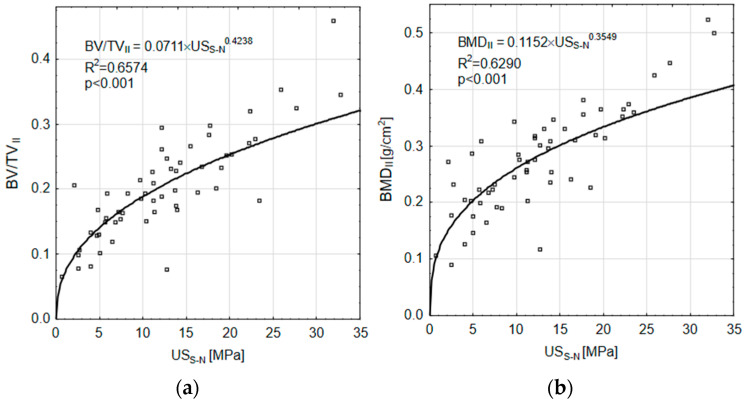
Relationship between structure indices and *US_S-N_*: (**a**) *BV/TV_II_*; (**b**) *BMD_II_*.

Relationships were established between the calculated *US_S-N_* compressive strength values and selected structural indicators for the Group II specimens. Figure 7a shows the *BV/TV_II_* values in relation to the *US_S-N_* compressive strength. For this relationship, the R^2^ = 0.6574 was obtained and the CI confidence interval ranged from 0.4935 to 0.7872; *p* < 0.001. Figure 7b shows the *BMD_II_* values in relation to the *US_S-N_* compressive strength. For this relationship, the R^2^ = 0.6290 was obtained and the CI confidence interval ranged from 0.4032 to 0.7349; *p* < 0.001.

The calculation relationships were used to indirectly evaluate the method used to determine the S-N curves from which the *US_S-N_* compressive strength values were calculated. Based on the relationship between the structure indices considered and the *US* compressive strength, relationships (4) were formulated to determine the calculated values of the *BV/TV_cal_* and *BMD_cal_* indices.

(4) $$\begin{array}{c} BV/{TV}_{cal}=0.0711\cdot {US}^{0.4236}\\ {BMD}_{cal}=0.1152\cdot {US}^{0.3549} \end{array}$$

The values of calculated indices *BV/TV_Ical_* and *BMD_Ical_* were determined using the calculation relationships (4) and the experimental values of ultimate compressive strength *US_I_* obtained for the Group I specimens (Table 5). Descriptive statistics of the calculated indices are presented in Table 8.

The agreement of the distributions of the values of *BV/TV_Ical_* and *BMD_Ical_* with the experimental values of *BV/TV_I_* and *BMD_I_* was examined statistically using the Kolmogorv–Smirnov test. The value of the statistic D and the *p*-value are shown in Table 9.

**Figure 8:**
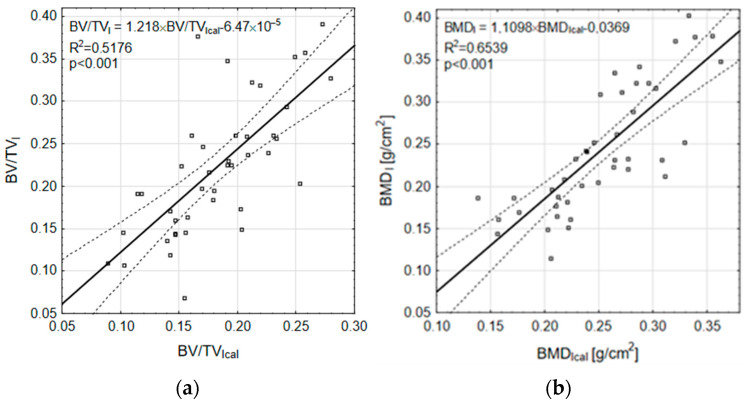
Correlation between calculated and experimental indices: (**a**) *BV/TV_I_*-*BV/TV_cal_*; (**b**) *BMD_I_*-*BMD_cal_*.

The *D* statistics and *p*-values indicate that there are no statistically significant differences between the distributions of *BV/TV_I_*-*BV/TV_Ical_* and *BMD_I_*-*BMD_Ical_*. The calculated values of the *BV/TV_I_* and *BMD_I_* indices were also compared with the experimental values using linear correlation plots. The graphical representation of this comparison is shown in Figure 8.

**Figure 9:**
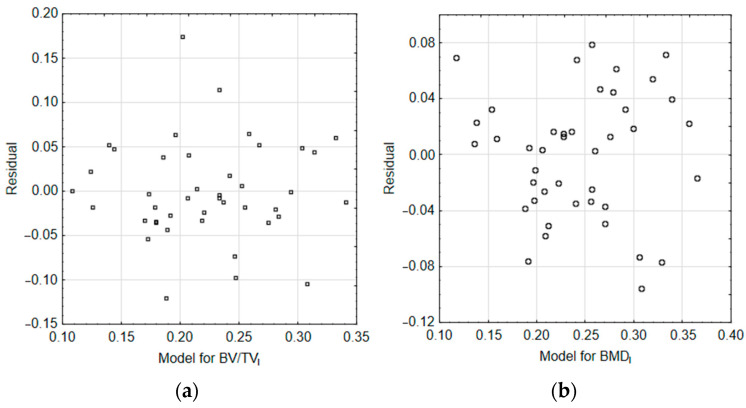
Plots of residuals for correlation between calculated and experimental indices: (**a**) *BV/TV_I_*-*BV/TV_Ical_*; (**b**) *BMD_I_*-*BMD_Ical_*.

For *BV/TV_I_*, the R^2^ = 0.5176 was obtained and the CI confidence interval ranged from 0.3164 to 0.7187; *p* < 0.001 (Figure 8a). For *BMD_I_*, the R^2^ = 0.6539 was obtained and the CI confidence interval ranged from 0.4916 to 0.8161; *p* < 0.001 (Figure 8b). The inner dotted lines in Figure 8 show the 95% confidence intervals. The residual plots for the correlations between calculated and experimental indices (Figure 8), shown in Figure 9, reveal no trend.

## 4. Discussion

The specimens used for this study have highly differentiated structures (Table 1). The difference between maximum *BV/TV* and minimum *BV/TV* is more than 577% for Group I and 697% for Group II. The corresponding *BMD* values are 351% and 577%, respectively. The range of variability of the *BV/TV* for both groups, i.e., 0.066–0.460, is comparable to or wider than the ranges used in other studies. Values of *BV/TV* indices in [17] were 0.039–0.218 for 29 cylindrical specimens obtained from human thoracic and lumbar vertebrae. In [34], *BV/TV* values ranged from 0.04 to 0.481 for 52 specimens obtained from the iliac crest, femoral head, second and fourth lumbar vertebrae and calcaneal core. In [50], the range was 0.05–0.35 in a study of 46 cylindrical specimens obtained from the human femoral neck, greater trochanter and vertebral body. The *BV/TV* reported in [51] was 0.15–0.34 for 15 specimens from the human femoral head, and 0.22–0.5 for 20 specimens from other bones: 10 of each from the human greater trochanter region and L-3 and L-4 vertebral bodies [52]. In [23], *BV/TV* was 0.123–0.296 for porcine vertebral bones. Similar analyses can also be performed for *BMD* obtained via different methods on 22 specimens from the human femoral head [53]. There is a statistical basis for the hypothesis that as regards the *BV/TV* values, both specimen groups are from the same population and the distribution is normal, and therefore the results can be transferred between groups. Not all statements regarding *BV/TV* can be transferred to *BMD*, as shown in [22,49], despite many indications in numerous studies on the golden standard in the diagnosis of, e.g., osteoporosis [54].

The first and most visible age-related change in the structure of trabecular bone is the loss of bone mass, expressed as a decrease in the mineral fraction described by the *BV/TV* index [55]. This effect is also clearly reflected in the decrease in *BMD* index [56]. The degradation of the trabecular structure, described by the decrease in the values of the microstructural parameters, is accompanied by an increase in the anisotropy of the structure described by the *DA* index [57]. In [58] it was shown that statistically significant changes in trabecular bone structure occur with age. A decrease in the value of the relative volume of trabecular bone (*BV/TV*) and the trabecular indices *Tb.N* and *Tb.Sp* was demonstrated via one-way ANOVA test. The indicated changes occurred mainly in middle age, with significant differences between the youngest and the two older age groups [58]. Age-related decreases in *BMD* are observed in both trabecular and cortical bone [59]. Decreased *BMD* may be a good predictor of hip fracture [60]. Changes in microstructure with age result in a decrease in bone strength properties [61]. The observed decrease in stiffness (Young’s modulus) for tibial trabecular bone begins after the age of 45, and the decrease in ultimate stress is already noticeable after the age of 40 [61]. Analysis of the bone’s response to fatigue damage also shows that the rate of damage increases with age [62]. Specimens in both groups were taken from individuals over the age of 45, when a decline in both mechanical properties and structural index values can be expected. The proposed verification method for fatigue limit determination combines *US_S-N_* with the *BV/TV* and *BMD* indices. As the changes in mechanical properties and the values of the selected indices follow the same decreasing trend, it can be assumed that the age distribution is not significant for the study.

The relationship between *US* and *BV/TV_I_* is not strong (R~0.7) and in some studies is considered higher [23,63]; however, the relationship was determined for 42 specimens from different donors. *BMD* features similar correlation coefficient, as shown in [64]. For the fatigue limit—maximum stress determined for stepwise increased load amplitude *σ_max_*—the relationship with *BV/TV_II_* gives the highest correlation coefficient of R > 0.8. This appears to be due to the greater sensitivity to cyclic load compared to monotonic load. This allows individual S-N curves to be plotted for each hypothetical sample group with the same or similar value of the coefficient describing the bone structure [17]. *BV/TV* can be used as such a coefficient. The calculation of a fatigue limit *σ_f_* using the Locati method for each specimen, assuming the same slope, gives an initial point of the plot. The end point corresponds to the *US_S-N_* compressive strength obtained for the *N* = 1 fatigue life.

The fatigue limit calculations were partially verified by the static loading experiment. For values of *US* introduced into the relationship *BV/TV_II_* vs. *US_S-N_*, the *BV/TV_cal_* values were calculated and compared to *BV/TV_I_*. A similar procedure was applied to the experimental and calculated values of the *BMD* indices. The experimental and calculated values were compared using linear correlation with 95% confidence intervals, as shown in Figure 8. If both values were in agreement, they would lie on the diagonal in the figure, but it can be seen that the calculated values are slightly higher. This it is due to the tendency of the model to overestimate the *US_S-N_* compressive strength.

The method presented in the paper assumes that the fatigue life *N_step_* is determined in tests with stepwise increasing load amplitude, for which the criterion used to determine specimen failure is important. The criterion used by the authors to determine specimen failure was an increase in specimen deformation greater than 10% of the median calculated for the last load level. In [65], cylindrical bone samples 6.7 mm in diameter and 8 mm in length were taken from the medial and lateral condyles of equine forelimbs. The specimens were stiffness adjusted to a stress of 23 MPa (20% of yield strength) and fatigue life was assessed via cyclic compression, loading the specimens from 0.8 to 70 MPa at a frequency of 2 Hz. During these tests, stiffness was calculated for each load cycle and the time of specimen failure was assumed to be the occurrence of the cycle with the lowest recorded stiffness. In [66], the right proximal tibiae of rats were tested after being placed in the moulds. The base length of the specimen was 8 mm. The specimens were subjected to cyclic loading at a frequency of 5 Hz between 0.1 N and the force F required to produce a strain of 20,000 με. The average of the first 10 cycles was taken as the initial stiffness. A 25% decrease in initial specimen stiffness was considered to be the time of specimen fatigue failure. In [67], cube-shaped samples taken from human tibia bone with a side of 4.5 mm were tested. The constant load amplitude fatigue test at a load corresponding to an initial strain of 5000 με was stopped when the target strain of 1000 με was reached in the specimen, but before fatigue failure occurred. Fatigue-preloaded specimens and unloaded control specimens containing microcracks were then tested at different strain rates.

The limitation of the presented method is mainly due to the assumptions made regarding the value of the initial modulus *E*_0_ and the slope coefficient b. In a situation where it was not possible to experimentally determine the S-N reference curve required by the Locati method due to the limited number of samples, a possible solution was to take it from the literature. However, these values may be subject to error because they were obtained from samples of bone structures other than those used in the study. The previously mentioned difference between the maximum and minimum values of *BV*/*TV* and *BMD* considered may be partly responsible for the scatter of results observed on linear correlation plots of the calculated values of the indices with the experimental data (Figure 8). Bearing in mind the above limitations, the regression models formulated in the paper, which describe the relationship between the fatigue limit σ_f_ and *BV/TV* and *BMD*, can be used with special caution in clinical practice to estimate *σ_f_* for bone structures whose indices have been determined in vivo.

According to the authors, the adopted modelling method can be further optimised by a critical analysis of the assumptions made for the model. The first subsequent area of research can be related to changing the values of the coefficients of the generalised material model adopted from the literature, which determines the position of the S-N curve. The second point to analyse is the change in the critical value of the accumulated damage described by the parameter *D* in Equation (1). The value of (1) used in this work is correct for some groups of metals, but may not be appropriate for bones.

## 5. Conclusions

This paper presents a method for determining the S-N curve for trabecular bone material from a specific donor. The proposed method of determining the position of this curve, based on the fatigue accumulation according to Miner’s law and the application of a stepwise increasing load amplitude during the fatigue test, allows the result to be obtained from a single bone sample. The proposed approach is characterised by a significant reduction in research time and workload compared to other methods of determining the S-N curve.

The paper presents an indirect verification of the method by comparing selected bone structure parameters obtained experimentally with those obtained computationally from the proposed relationships. The obtained values of R^2^ determination index values of 51% for *BV/TV* and 65% for *BMD* do not prove the high statistical power of the method, but it should be noted that they were obtained with a gain close to 1 and a bias of approximately 0, as seen in Figure 8. Furthermore, it is worth noting that similar R^2^ values are also obtained for the relationships relating *σ_f_* to the *BV/TV* and *BMD* structural indicators (Figure 5). Therefore, it is possible that the error in the presented method is at least partly induced by errors in these relationships. Due to the indirect method of model verification used, it was not possible to determine the significance level of this effect.

## Figures and Tables

**Table 1:** Descriptive statistics of patients.

		Grup I	Grup II
Number of samples	*n_total_*	42	55
	*n_male_*	14	18
	*n_female_*	28	37
	*n_m_*/*n_f_*	0.5	0.49
Age of patients	Min	50	46
	Max	91	88
	Mean	73.0	72.6
	SD	8.42	8.74

**Table 2:** Descriptive statistics of selected structural indices.

Samples	Indicator	Unit	Min	Max	Mean	SD
Group I	*BV/TV_I_*	[-]	0.068	0.392	0.222	0.079
	*BMD_I_*	[g/cm^2^]	0.115	0.404	0.243	0.075
Group II	*BV/TV_II_*	[-]	0.066	0.46	0.203	0.077
	*BMD_II_*	[g/cm^2^]	0.091	0.525	0.276	0.091

**Table 3:** Shapiro–Wilk test of selected structural indices (*p* > 0.05).

Samples	Indicator	Unit	*W*-Statistic	*p*-Value
Group I	*BV/TV_I_*	[-]	0.968	0.298
	*BMD_I_*	[g/cm^2^]	0.946	0.046
Group II	*BV/TV_II_*	[-]	0.97	0.327
	*BMD_II_*	[g/cm^2^]	0.977	0.558

**Table 4:** Kolmogorov–Smirnov test of selected structural indices (*p* > 0.05).

Tested Pair		*D*-Statistic	*p*-Value
*BV/TV_I_*	*BV/TV_II_*	0.119	0.927
*BMD_I_*	*BMD_II_*	0.381	0.005

**Table 5:** Descriptive statistics of mechanical tests results.

Samples	Property	Unit	Min	Max	Mean	SD
Group I	*US*	[MPa]	1.678	25.288	10.206	5.85
Group II	*σ_max_*	[MPa]	0.397	12.92	5.3	2.92
	*N_step_*	[cycle]	1185	50,535	20,656	11,623

**Table 6:** Shapiro–Wilk test of experiment results.

Samples	Property	Unit	*D*-Statistic	*p*-Value
Group I	*US*	[MPa]	0.939	0.026
Group II	*σ_max_*	[MPa]	0.951	0.023
	*N_step_*	[cycle]	0.961	0.072

**Table 7:** Descriptive statistics of calculation results (Group II).

Property	Unit	Min	Max	Mean	SD
*σ_f_*	[MPa]	0.101	5.552	2.112	1.277
*US_S-N_*	[MPa]	0.5954	32.69	12.44	7.52

**Table 8:** Descriptive statistics of calculated structural indices (Group I).

Indicator	Unit	Min	Max	Mean	SD
*BV/TV_Ical_*	[-]	0.089	0.28	0.182	0.047
*BMD_Ical_*	[g/cm^2^]	0.138	0.363	0.252	0.054

**Table 9:** Kolmogorov–Smirnov test for experimental and calculated structural indices.

Tested Pair		*D*-Statistic	*p*-Value
*BV/TV_I_*	*BV/TV_Ical_*	0.286	0.065
*BMD_I_*	*BMD_Ical_*	0.238	0.185

## Data Availability

The raw data supporting the conclusions of this article will be made available by the authors on request due to its inclusion in an ongoing study.
